# Interleukin-6/STAT3 signalling regulates adipocyte induced epithelial-mesenchymal transition in breast cancer cells

**DOI:** 10.1038/s41598-018-27184-9

**Published:** 2018-06-11

**Authors:** Jones Gyamfi, Yun-Hee Lee, Minseob Eom, Junjeong Choi

**Affiliations:** 1 College of Pharmacy, Yonsei Institute of Pharmaceutical Sciences, Yonsei University, Incheon, Korea; 2 Department of Pathology, Yonsei University Wonju College of Medicine, Wonju, Korea

**Keywords:** Cancer microenvironment, Mechanisms of disease, Breast cancer

## Abstract

The tumour microenvironment is a key regulators of tumour progression through the secretion of growth factors that activate epithelial-mesenchymal transition (EMT). Induction of EMT is a key step for transition from a benign state to a metastatic tumour. Adipose tissue forms a bulk portion of the breast cancer microenvironment, emerging evidence indicates the potential for adipocytes to influence tumour progression through the secretion of adipokines that can induce EMT. The molecular mechanisms underlying how adipocytes enhance breast cancer progression is largely unknown. We hypothesized that paracrine signalling by adipocytes can activate EMT and results in increased migration and invasion characteristics of breast cancer cells. We found that IL-6 secreted by adipocytes induce EMT in breast cancer cells. The effect of IL-6 expression on EMT is mediated through activation of the signal transducer and activated of transcription 3 (STAT3). Blocking of IL-6 signalling in breast cancer cells and adipocytes, decreased proliferation, migration and invasion capabilities and altered the expression of genes regulating EMT. Together, our results suggest that matured human adipocytes can enhance the aggressive behaviour of breast cancer cells and induce an EMT-phenotype through paracrine IL-6/STAT3 signalling.

## Introduction

Breast cancer is the most common malignancy and a leading cause of cancer-related death in women worldwide^1^. Presently, treatment and management of breast cancer has significantly improved with increased understanding to the disease biology. In breast cancer, the tumour microenvironment is dominated by stromal cells such as fibroblasts, endothelial cells, immune cells and adipocytes^2,3^. Recent studies have focused on elucidating the mechanisms by which growth factors secreted by components of the tumour microenvironment enhance tumour progression. Epithelial-mesenchymal transition (EMT) is a key process hypothesized to be activated by tumour microenvironment that enhance tumour procession. Epithelial-mesenchymal transition (EMT) is a well-documented molecular event that enhance invasion and metastasis of breast cancer cells^4,5^. The EMT process in cancer cells is characterised by tumour epithelial cells undergoing molecular and genetic changes resulting in the loss of cell junctions, apical-basal polarity, acquisition of a mesenchymal phenotype with enhanced migration and invasion potential^6^. Epithelial cells express specific proteins such as E-cadherin’s, occludins and cytokeratin; however, during EMT, epithelial cells decrease expression of E-cadherin and increase the expression of mesenchymal phenotype specific proteins such as N-cadherin’s and vimentin^6,7^. Regulation of EMT is associated with aberrant expression of usually repressed transcriptional factors such as snail homolog 1 (SNAIL), twist basic helix-loop-helix transcription factor (TWIST), FOXC2, ZEB1 and ZEB2^8^. In breast cancer, the EMT phenotype is associated with increased cell motility, invasion and enhanced metastasis^9^. The components of the tumour microenvironment have emerged as key contributors to tumourigenesis. Paracrine interaction between stromal and breast cancer cells have been shown to enhance the metastatic potential breast cancer cells^2,3^. Several signalling pathways activated in the tumour microenvironment are essential regulators of EMT^7,10,11^.

Adipose tissues are the most abundant tissues in the breast cancer microenvironment, initially regarded as providing support, insulation and serving as site for energy storage^12,13^. The potential for adipocytes to influence breast cancer cells migration and invasion, and ultimately result in metastasis has begun to emerge^12,14,15^. With various studies focused on determining how paracrine signalling by adipocytes enhance breast cancer progression. The secretion of hormones, growth factors and cytokines (collectively referred to as adipocytokines) by adipocytes have been hypothesized to activate various signalling pathways in the nearby tumour cells resulting in increased migration and invasion in breast cancer cells^16^. Among the growth factors secreted by adipocytes, transforming growth factor-beta (TGF-β) and interleukin-6 (IL-6) have been independently proven to be potent regulators of EMT in various cancer cells^7,10,17,18^. TGF-β through the SMADs transcription factors can induce EMT, invasion and migration in epithelial cells and breast cancer cells^18,19^. The pleotropic cytokine, IL-6 is highly expressed in adipose tissue and play a multifactorial role in cancer, influencing EMT, metastasis, angiogenesis, cachexia, stemness and therapeutic resistance^20–22^. Addition of synthesized IL-6 to breast cancer cells was demonstrated to induced EMT via activation of the signal transducer and activated of transcription 3 (STAT3)^7,23^. A recent study indicate that adipocytes can enhance invasiveness and induce EMT in breast cancer cells^12,24^. However, the molecular mechanisms by which adipocyte induce EMT in breast cancer cells occurs has not been widely explored.

In this study, we investigated the molecular mechanism by which human adipocytes influence proliferation, invasion and migration capabilities of breast cancer cells of different characteristics. We found that adipocytes enhanced proliferation, migration and invasion characteristics of breast cancer cells with the emergence of an EMT phenotype. Adipocytes and breast cancer cells co-cultured had increased expression of IL-6. Moreover, we found that induction of EMT in breast cancer cells involved phosphorylation and nuclear localization of STAT3. Hence, blocking IL-6 signalling partially reversed the EMT phenotype and decreased the proliferation, migration and invasion characteristics of breast cancer cells. Our data demonstrates that human adipocytes can enhance the proliferation, invasion and migration characteristics of breast cancer cells and induce an EMT phenotype through paracrine IL-6/STAT3 signalling.

## Results

### Co-culture with human adipocytes enhanced proliferation, migration and invasion in breast cancer cells

**Figure 1:**
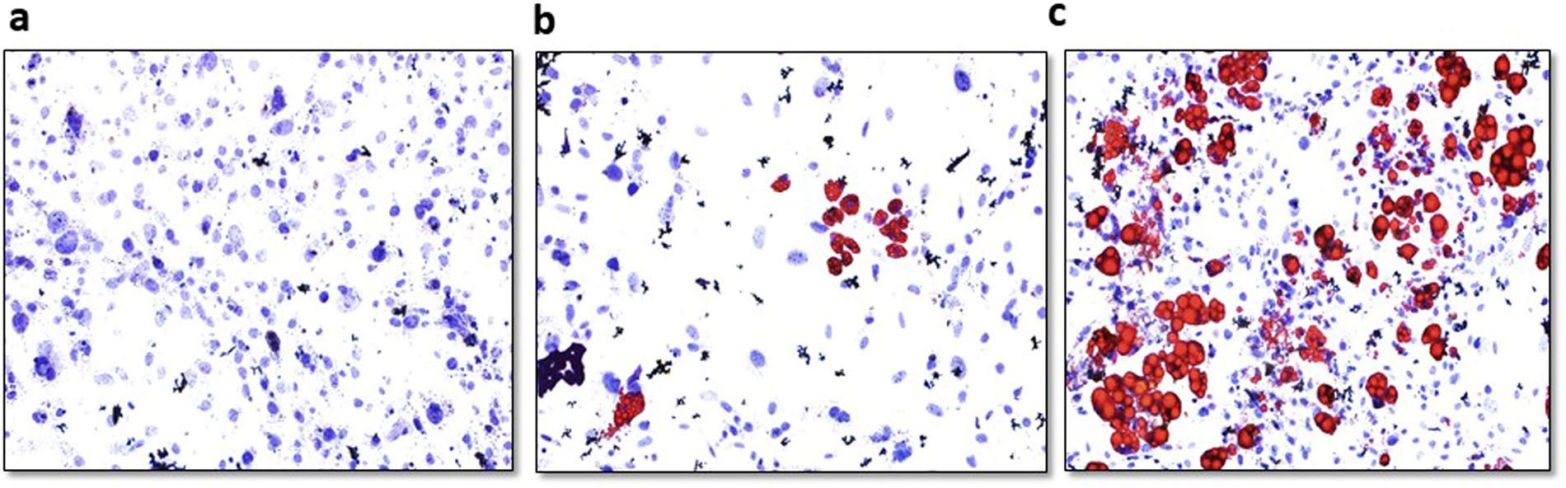
Oil Red O staining of differentiated human preadipocytes. (**a**) Undifferentiated human preadipocytes, 4 weeks after culturing without induction media. (**b**) Differentiating human preadipocytes with tiny lipid droplets 2 weeks after induction of differentiation. (**c**) Differentiated human preadipocytes 4 weeks after induction of differentiation with large formed lipid droplets.

**Figure 2:**
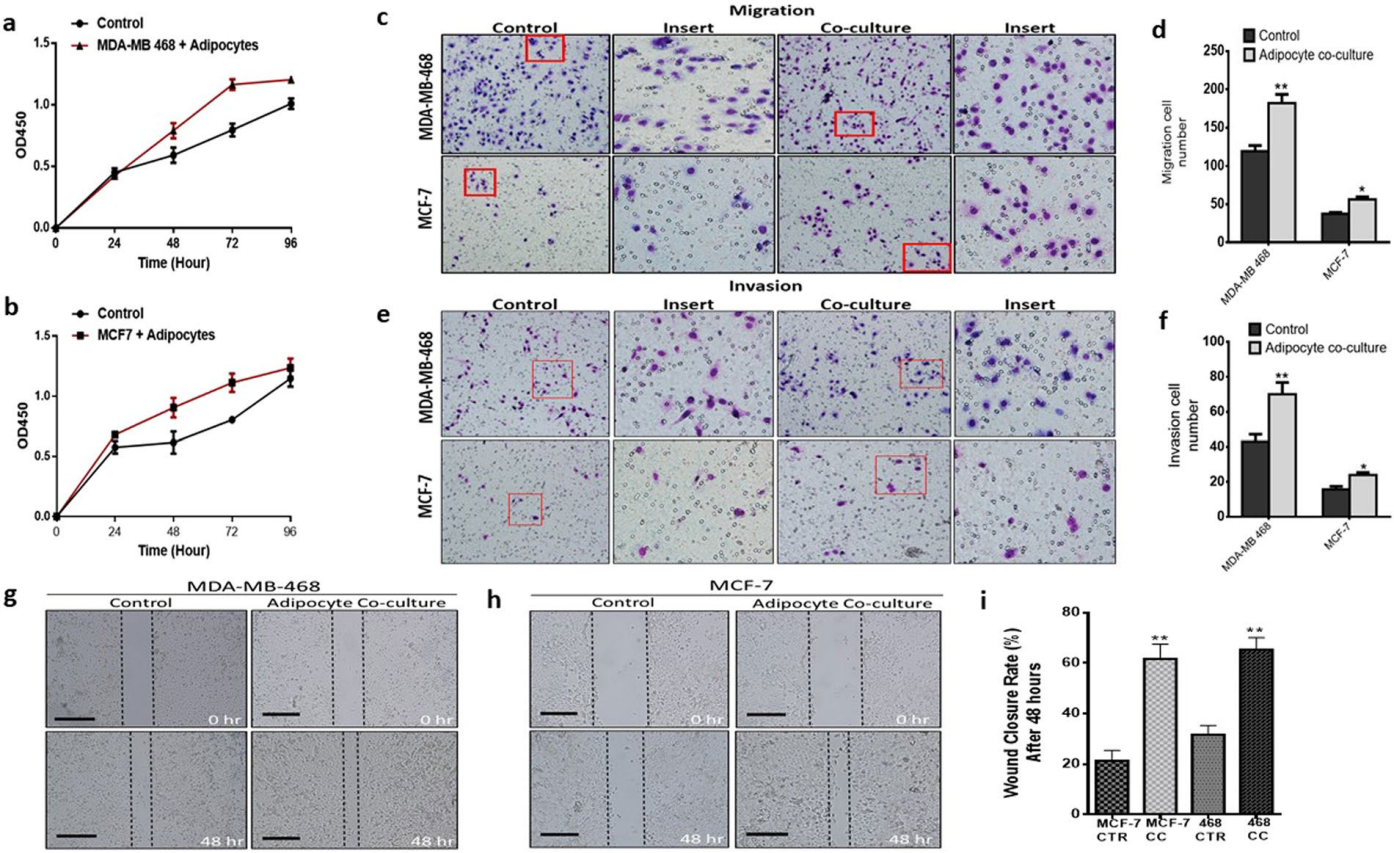
Human adipocytes enhances the aggressive characteristics of breast cancer. (**a,b**) Proliferation capabilities of co-cultured breast cancer cells assessed with the CCK-8 assay, compared with control cells (A: MDA-MB-468 and B: MCF-7). (**c**) Migration rate was examined using the transwell insert without matrigel. (**d**) Quantification of migration capabilities ability of MDA-MB-468 and MCF-7 breast cancer cells. (**e**) Invasion rate was examined using the transwell insert with matrigel. (**f**) Quantification of invasion capabilities ability of MDA-MB-468 and MCF-7 breast cancer cells. (**g,h,i**) Representative images of wound healing assay. Breast cancer cell motility accessed by a wound healing assay after 48 hours (magnification, x100), MDA-MB-468 and MCF-7 co-cultured breast cancer cells had a higher motility rates. All results are representative of 3 independent experiments. (Data indicate mean ± SD; ***p < 0.001; **p < 0.01; *p < 0.05).

To mimic the breast cancer stromal microenvironment and explore how human adipocytes influence breast cancer cell behaviour, we used the transwell co-culture system, where differentiated human adipocytes was co-cultured with breast cancer cells. Adipocytes were differentiated in the bottom of the transwell 6-well culture plate (Fig. 1) and breast cancer cells were seeded into the co-culture insert. The co-culture system was used to investigate the effect of differentiated mature human adipocytes on the proliferation of breast cancers cells with different molecular phenotypes; MDA-MB-468 (triple negative) and MCF-7 (luminal). Co-culture with human adipocytes increased the proliferation rate of MDA-MB-468 and MCF-7 breast cancer cells compared to control cells (p < 0.001) (Fig. 2a,b). The transwell co-culture system with and without matrigel was used to investigate the effect of adipocytes on breast cancer cell migration and invasion characteristics. Compared to control cells, breast cancer cells co-cultured with adipocytes also had increased migration (p < 0.05) (Fig. 2c,d) and invasion (p < 0.05) (Fig. 2e,f) characteristics. Our results demonstrate the potential for human adipocytes to enhance proliferation, migration and invasion abilities of MDA-MB-468 and MCF-7 cells after co-culture. These effects were more profound in MDA-MB-468 cells compared with MCF-7, which are non-invasive cells. To investigate the effect of adipocyte on the motility capability, MDA-MB-468 and MCF-7 cells co-cultured with adipocytes were grown to a confluent monolayer and wounded longitudinal. Cells are incubated for an appropriate time and images taken at 0 and 48 hours. Compared with control cells, both MDA-MB and MCF-7 cells co-cultured with adipocytes had an increased motility and an increased wound closure rate (p < 0.001) (Fig. 2g,h,i). These finding are consistent with previous studies where adipocyte conditioned media or murine adipocytes stimulated increased proliferation, migration and invasion in breast cancer cell^13,14,24^. Collectively, these results indicate that human adipocytes can enhance the proliferation, migration and invasion capabilities of MDA-MB-468 and MCF-7 breast cancer cells.

### Human adipocytes induced an EMT-phenotype with expression of EMT-related genes in breast cancer cells

**Figure 3:**
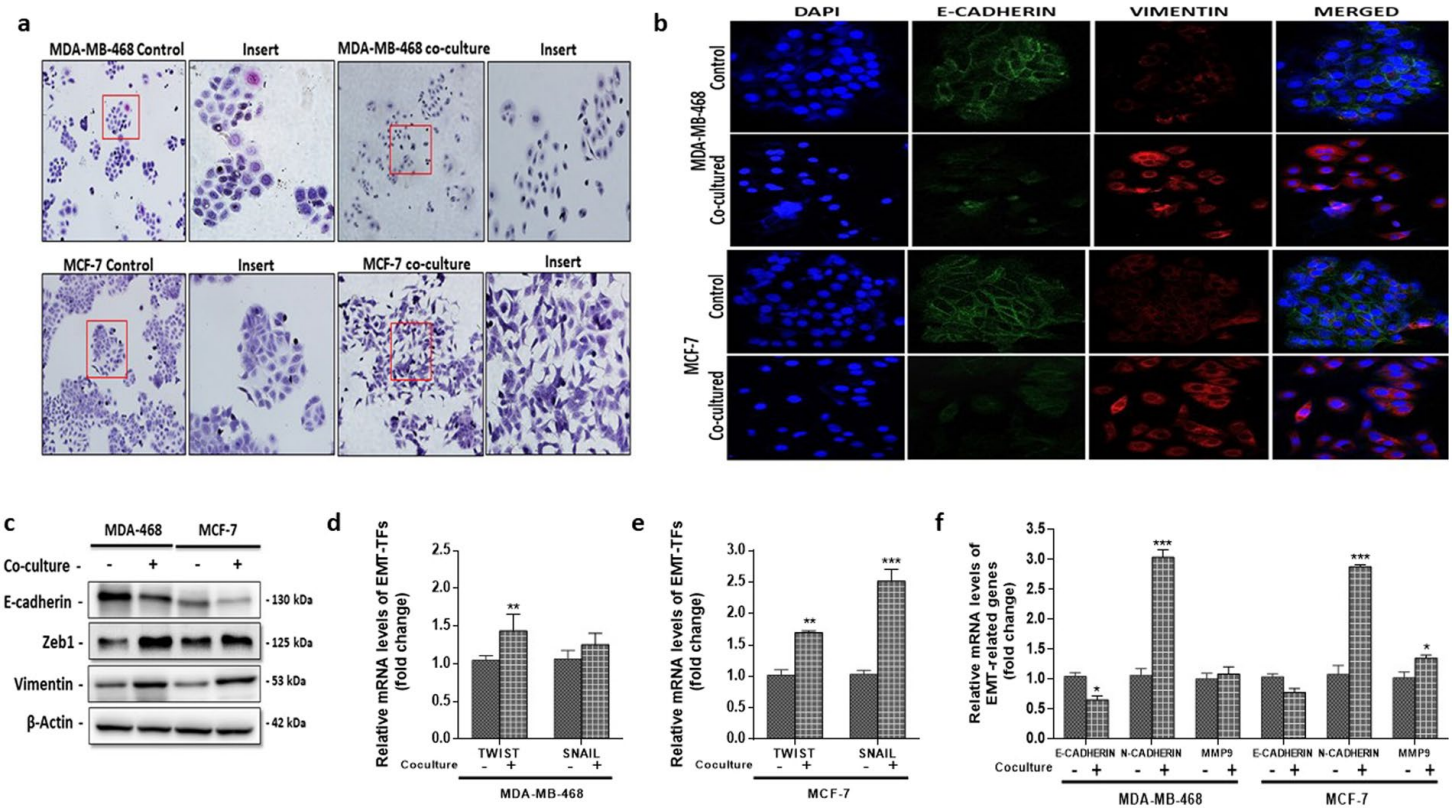
Human adipocytes induced EMT-phenotype with alteration in EMT-related gene expression. (**a**) Morphological characteristics of MDA-MB-468 and MCF-7 cells compared with cells co-cultured with human adipocytes. Co-cultured breast cancer cells were dispersed and slightly elongated. (**b**) Immunofluorescence staining of e-cadherin and vimentin. (**c**) Western blot for e-cadherin, vimentin and ZEB1 in MDA-MB-468 and MCF-7 breast cancer cells cultured with or without adipocytes. Full-length blots are presented in Supplementary Fig. 5. (**d,e**) Quantitative PCR (qt-PCR) comparing the expression of EMT-TFs (TWIST and SNAIL) in co-cultured breast cancer cells to control cells. (**f**) qt-PCR comparing the expression of EMT-related genes (MMP9, N-cadherin and E-cadherin) in co-cultured breast cancer cells to control cells. Relative mRNA expression was normalized to GADPH. All results are representative of 3 independent experiments. (Data indicate mean ± SEM; ***p < 0,001; **p < 0.01; *p < 0.05).

Epithelial-mesenchymal transition is linked to the increased proliferation, migration, invasion and metastasis of breast cancer cells (see review^9^). Adipocytes have been shown to be capable of inducing EMT in breast cancer cells, hence enhancing their proliferation, migration and invasion. To investigate the mechanism by which co-cultured human adipocytes induced aggressive behaviour occur in breast cancer cells, we determined if changes the expression of EMT-related proteins and genes in MDA-MB-468 and MCF-7 cells could count for the enhanced aggressive behaviour. Epithelial MDA-MB-468 and MCF-7 co-cultured with adipocytes were more dispersed and showed a slightly elongated morphology compared to control cells (Fig. 3a). Cells in EMT have been reported to have an elongated phenotype with changes in the expression levels of e-cadherin and vimentin. We analysed the expression of EMT-related proteins (E-cadherin and vimentin) by immunofluorescence. We observed that in both MDA-MB-468 and MCF-7 cells cultured with adipocytes, the cells became dispersed with a decreased expression of E-cadherin (>50% decrease) and an increased expression of vimentin (>50% increase) (Fig. 3b). Changes in the expression of EMT proteins were confirmed by western blot. Both MDA-MB-468 and MCF-7 showed a decrease expression in the epithelial cell marker E-cadherin and in cells after co-culture and an increase in mesenchymal cell marker vimentin (Fig. 3c). Expression of ZEB1 a marker for EMT was also increased in both cells after co-culture with adipocytes. These finding suggest that the presence of adipocytes can induced an EMT-phenotype in epithelial breast cancer cells.

EMT is cancer cells is linked to changes in the expression of EMT-transcription factors (EMT-TFs) such as SNAIL and TWIST and EMT-related genes such as E-cadherin, N-cadherin and matrix metalloprotease 9 (MMP9). To confirm if the observed EMT-phenotype is regulated by changes in the expression of EMT-related genes. We examined the expression of EMT-TFs (SNAIL and TWIST) and EMT-related genes (E-cadherin, N-cadherin and MMP9) in breast cancer cells co-cultured with adipocytes by quantitative real time PCR (qtPCR). Our results were consistent with changes associated with EMT, the EMT-TFs SNAIL and TWIST were upregulated in both MDA-MB-468 and MCF-7 cells (Fig. 3d,e), whereas E-cadherin expression was downregulated **(**Fig. 3f**)**. The expression of EMT-related gene N-cadherin was significantly upregulated in both cells (Fig. 3d,e), but MMP9 expression was not significantly upregulated. Collectively, these findings indicate that the presence of adipocytes may have the potential to induce an EMT-phenotype in breast cancer cells via activation of different EMT-related genes in different breast cancer cells.

### Adipocytes activated IL-6/STAT3 signalling in co-cultured breast cancer cells

**Figure 4:**
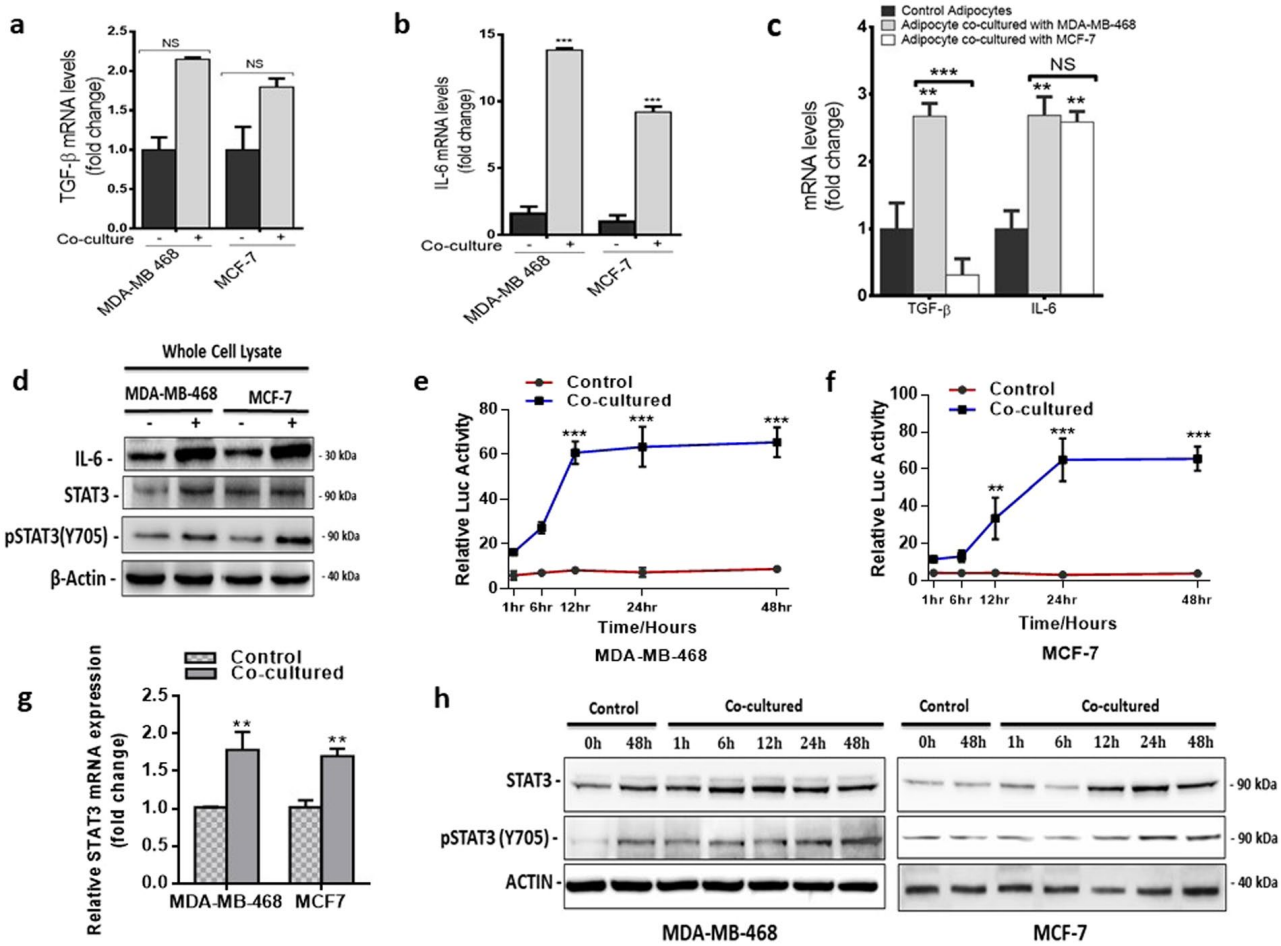
Analysis of STAT3 activity in breast cancer cells. (**a,b**) Quantitative PCR comparing the expression of TGF-B and IL-6 in co-cultured breast cancer cells to control cells. (**c**) qt-PCR comparing the expression of TGF-B and IL-6 in adipocytes cultured with/without breast cancer cells. (**d**) Comparing the expression levels of IL-6, STAT3 and pSTAT3 (Y705) in co-cultured and control breast cancer cells by immunoblotting. Full-length blots are presented in Supplementary Fig. 5. (**e,f**) Relative luciferase activity in breast cancer cells with STAT3 luciferase reporter plasmid and cultured with/without human adipocytes (**e**). MDA-MB-468 STAT3 activity, (**f**). MCF-7 STAT3 activity. Luciferase activity was normalized against a non-inducible luciferase construct. (**i**) Western blot for STAT3 and pSTAT3 (Y705) over 48 hours are regular time intervals in breast cancer cells cultured with/without adipocytes. B-actin was used as loading control. Full-length blots are presented in Supplementary Fig. 5. All results are representative of 3 independent experiments. Data is presented as mean ± SEM; ***p < 0,001; **p < 0.01; *p < 0.05.

Paracrine and endocrine signalling mechanism has been identified as a key mechanism by which adipocytes influence breast cancer cells in the tumour microenvironment^13^. The release of various growth factors by adipocytes to influence tumour proliferation, invasion and migration^12^. To identify paracrine regulator of EMT in adipocyte and breast cancer cell interaction, we compared mRNA expression of TGF-β and IL-6 (two prominent inducers of EMT) in breast cancer cells. Our results showed the level of both TGF-β and IL-6 was increased in MDA-MB-468 and MCF-7 cells cultured with adipocytes (p < 0.05) (Fig. 4a,b). IL-6 levels were increased in adipocytes cultured with both breast cancer cells, whereas TGF-β levels was increased only in adipocytes cultured with MDA-MB-468 cells but not with MCF-7 cells (Fig. 4c). Thus, we speculate that IL-6 maybe a common regulating cytokine in adipocyte-breast cancer cell interaction. Previous studies have shown that activation of IL-6 induces phosphorylation and activation of STAT3 transcription factors that regulate EMT-related genes (TWIST and MMP9) associated with EMT. To determine if adipocytes induced proliferation, migration and invasion in breast cancer cells occur by a similar mechanism, we compared the expression of IL-6, STAT3 and phosphorylated STAT3 (Y705) in breast cancer cells co-cultured with adipocytes (Fig. 4d). Co-culture with adipocytes increased in IL-6 and phosphorylated STAT3 expression in both MDA-MB-468 and MCF-7 cells compared to control cells, whereas STAT3 expression remained stable. As expected the expression of IL-6 was lower in MCF7 cells compared to MDA-MB-468, however, the levels of phosphorylated STAT3 was similar in both cells. These results indicate the IL-6/STAT3 signalling pathway to be activated in co-cultured breast cancer cells.

To further explore the role of the IL-6/STAT3 signalling pathway, we generated a stable MDA-MB-468 and MCF-7 cell line expressing a STAT3 reporter. Allowing STAT3 activity to be monitored by luciferase assay, we examine the activity of STAT3 over 48 hours at regular time intervals (1, 6, 12, 24 and 48 hours). In control cells, the activity levels of STAT3 remained fairly stable over the time period, transiently increasing and decreasing at various time but with no significant increase in STAT3 activity over the time period (p > 0.05) (Fig. 4e,f). In co-cultured MDA-MB-468 cells, STAT3 activity increased stably peaking within 6 hours of co-culture, and remained constitutively active for the rest of the period (p < 0.01) (Fig. 4e). In co-cultured MCF-7 cells, STAT3 activity was increased stably over the first 12 hours (p > 0.05) peaking after 12 hours and remained active over the remaining 36 hours (p < 0.01) (Fig. 4f). Activated STAT3 activity was confirmed by western blot for STAT3 and pSTAT3. Western blots finding confirmed luciferase results, where in MDA-MB-468 cells a stable increase of STAT3 activity was observed after 6 hours of co-culture. Increase in pSTAT3 expression was observed at 24 and 48 hours (Fig. 4h). In co-cultured MCF-7, increase in STAT3 expression occurred at 12 hours after co-culture, with pSTAT3 activity increasing at 24 and 48 hours (Fig. 4h). These finding were confirmed by increased STAT3 mRNA expression after co-culture with adipocytes (p < 0.01) (Fig. 4g). Taken together, these finding indicate that human adipocytes activated IL-6/STAT3 signalling in breast cancer cells. With increase STAT3 activity occurring within 6 hours of co-culture of breast cancer cells with adipocytes, this results in STAT3 phosphorylation and activation.

### Adipocytes induced nuclear localization of phosphorylated STAT3

**Figure 5:**
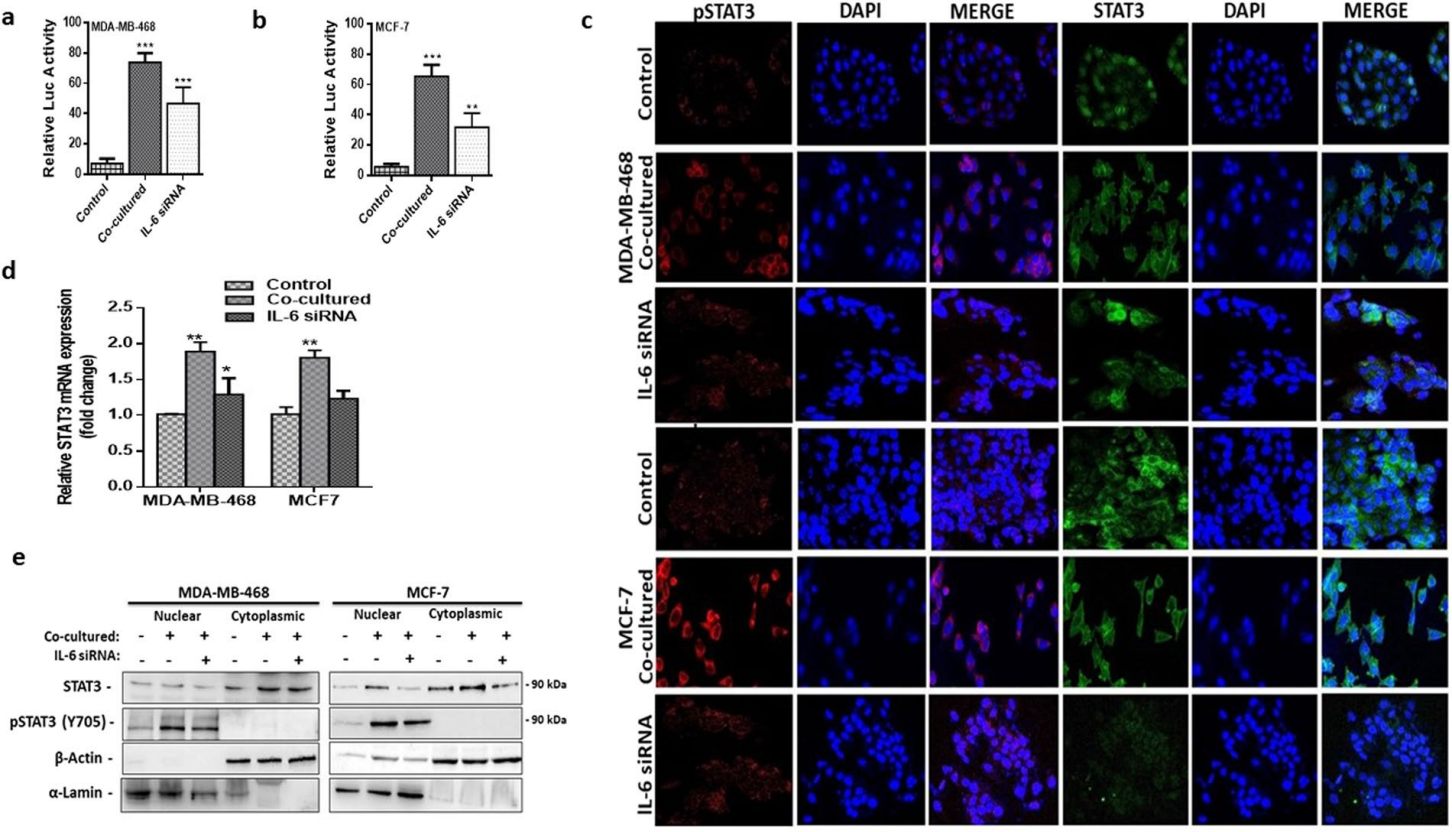
Adipocytes induces phosphorylated STAT3 nuclear localization. Using IL-6R siRNA and IL-6 neutralizing antibodies paracrine IL-6 signaling was blocked in co-cultured cells. (**a**) IL-6 mRNA expression was analyzed 72 hours after IL-6 signal was blocked. (**b,c**) Western blot for IL-6 in breast cancer cells over 72 hours after IL-6 signal was blocked at 24, 48 and 72 hours. Full-length blots are presented in Supplementary Fig. 5. (**d**) IL-6 mRNA expression was analyzed at 24, 48 and 72 hours in adipocytes after IL-6 siRNA transfection. (**e**) Western blot for IL-6 in IL-6 siRNA transfected adipocytes at 24, 48 and 72. Full-length blots are presented in Supplementary Fig. 5. (**f,g**) Relative luciferase activity in breast cancer cells with STAT3 luciferase reporter plasmid, with/without human adipocytes and after IL-6 was blocked. (**h**) Quantitative PCR comparing the expression of STAT3 mRNA in co-cultured breast cancer cells with/without IL-6 blocking and in control cells. Relative mRNA expression was normalized to GAPDH. (**i**) Increased STAT3 phosphorylation and nuclear localization in co-cultured human breast cancer cells with/without IL-6 neutralization and in control cells assessed by immunofluorescence staining. (**j**) Representative western blot analysis of pSTAT3 expression in cytoplasmic and nuclear fraction of co-cultured breast cancer with/without IL-6 neutralization and in control breast cancer cells. α-Lamin and β-actin was used as loading control for nuclear and cytoplasmic fraction respectively. Full-length blots are presented in Supplementary Fig. 5. All results are representative of 3 independent experiments. Data is presented as mean ± SEM; ***p < 0,001; **p < 0.01; *p < 0.05.

To better understand the paracrine IL-6/STAT3 signalling between adipocytes and breast cancer cells. We knockdown IL-6 in both adipocytes and breast cancer cells with an siRNA. Knockdown efficiency was examined by qtPCR and western blot, our results showed that IL-6 siRNA reduced IL-6 expression by over 90% during the first 72 hours in breast cancer cells (Fig. 5a–c), whiles in adipocytes IL-6 siRNA reduced IL-6 expression by about 50% with 24 hours (Fig. 5d,e) but expression of IL-6 increased with 48 hours. To ensure complete inhibition of secreted IL-6, we added neutralising IL-6 antibody to the co-culture system for subsequent experiments. Using a stable STAT3 reporter breast cancer cells, we observed the activity of STAT3 decreased after IL-6 signal was blocked in breast cancer cells (Fig. 5f,g). We confirm the decrease STAT3 activity by analysing STAT3 mRNA expression, STAT3 mRNA levels decreased after IL-6 signalling was blocked. Thus, blocking of IL-6R in breast cancer cells and neutralization of secreted IL-6 decreased STAT3 activity in breast cancer cells.

Given the consistent activation of STAT3 and increased phosphorylation of STAT3 signalling in co-cultured breast cancer cells we speculate that phosphorylated STAT3 drive the expression of EMT-related genes in co-cultured breast cancer cells. To explore this mechanism, we examined the localization of pSTAT3 in in co-cultured adipocytes. Immunofluorescence staining confirmed the activation of STAT3 signalling in co-cultured breast cancer cells, with increased levels of pSTAT3 (>20% increase). P-STAT3 also localizes in the nucleus of co-cultured cells (Fig. 5i). Western blot of cytoplasmic and nuclear fraction showed an increased expression of pSTAT3 in nuclear fraction of co-cultured cells than in nuclear fraction of control and IL-6-blocked breast cancer cells (Fig. 5j). Taken together, these results demonstrate that blocking IL-6 decrease STAT3 activity and phosphorylated STAT3 localises in the nucleus in co-cultured cells.

### Blocking IL-6 signalling decreases the proliferation, migration and invasion capabilities of co-cultured breast cancer cells

**Figure 6:**
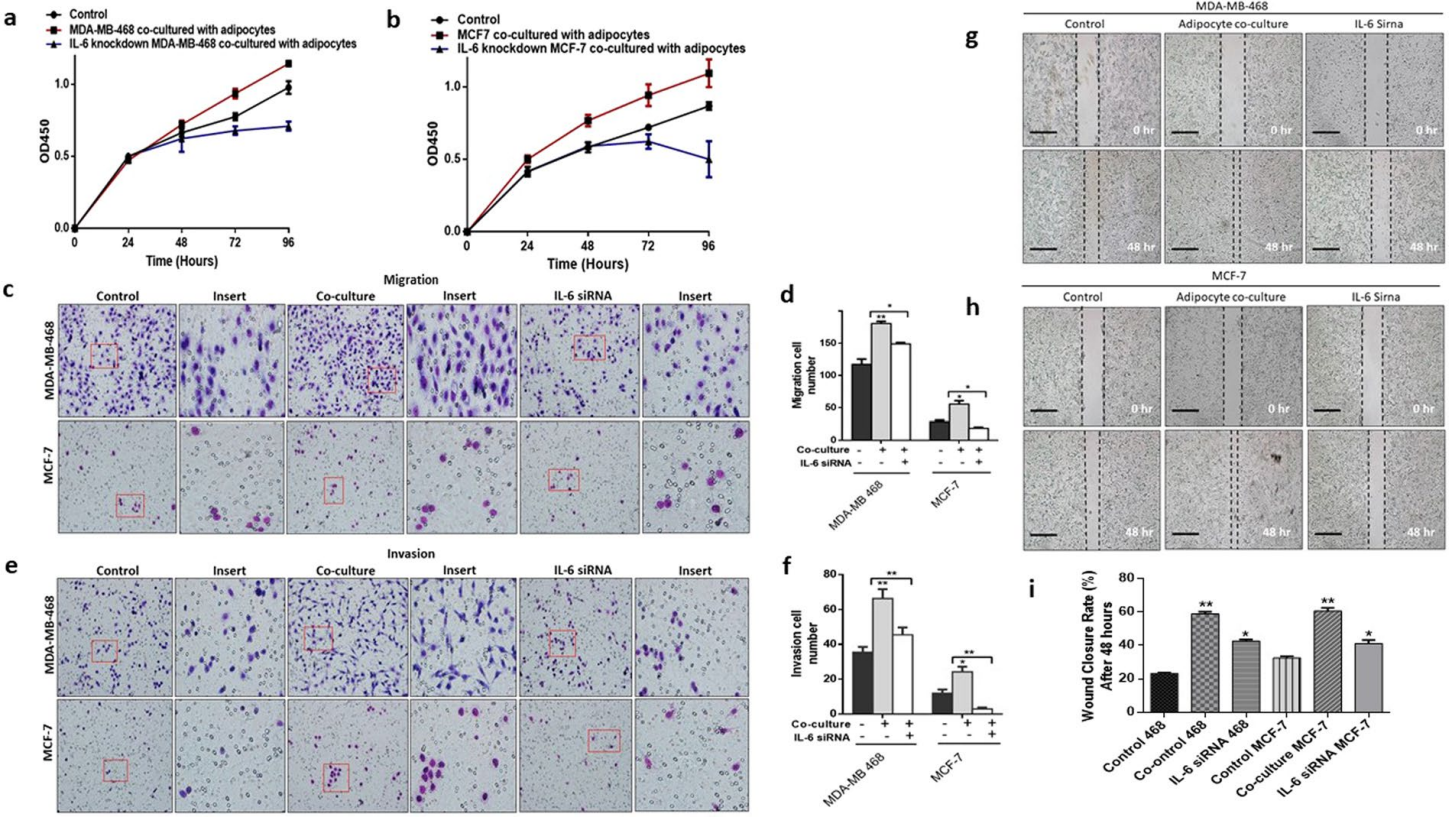
Blocking IL-6 signals reverses human adipocytes induced aggressive characteristics of breast cancer. (**a,b**) Proliferation capabilities of co-cultured breast cancer cells assessed in co-cultured breast cancer cells with/without IL-6 neutralization and in control cells. (**c**) Migration rate was examined using the transwell insert without matrigel in co-cultured breast cancer cells with/without IL-6 neutralization and in control cells. (**d**) Quantification of migration capabilities ability of in co-cultured breast cancer cells with/without IL-6 neutralization and in control cells. (**e**) Invasion rate in co-cultured breast cancer cells with/without IL-6 neutralization and in control cells. (**f**) Quantification of invasion capabilities in co-cultured breast cancer cells with/without IL-6 neutralization and in control cells. (**g, h**) Representative images of wound healing assay. Breast cancer cell motility accessed by a wound healing assay after 48 hours (magnification, x100), MDA-MB-468 and MCF-7 co-cultured breast cancer cells had a higher motility rates and blocking IL-6 inhibited breast cancer cell motility. (**i**) Quantification of breast cancer cell motility in co-cultured breast cancer cells with/without IL-6 neutralization and in control cells. All results are representative of 3 independent experiments. (Data indicate mean ± SD; ***p < 0.001; **p < 0.01; *p < 0.05).

To better understand the effect of paracrine IL-6 signalling in adipocyte-breast cancer interaction we determined the proliferation, migration and invasion characteristics of breast cancer cells in co-culture system after IL-6 signalling was blocked. Blocking of IL-6 signalling reversed the initial increase in proliferation, migration, and invasion characteristics induced by adipocyte co-culture. Both MDA-MB-468 and MCF-7 breast cancer cells had decreased proliferation (p < 0.01) (Fig. 6a,b), migration (p < 0.05) (Fig. 6c,d) and invasion (p < 0.01) (Fig. 6e,f) characteristics. The increased wound closure rate in co-cultured breast cancer cells was decreased significantly (p < 0.05) (Fig. 6g–i) after blocking IL-6 signalling. Taken together, these data indicate that blocking paracrine IL-6 signalling between breast cancer and adipocytes significantly inhibit the proliferation, migration and invasive capabilities of MDA-MB-468 and MCF-7 breast cancer cells.

### EMT-phenotype and gene expression were reversed by blocking IL-6 signalling in breast cancer cells

**Figure 7:**
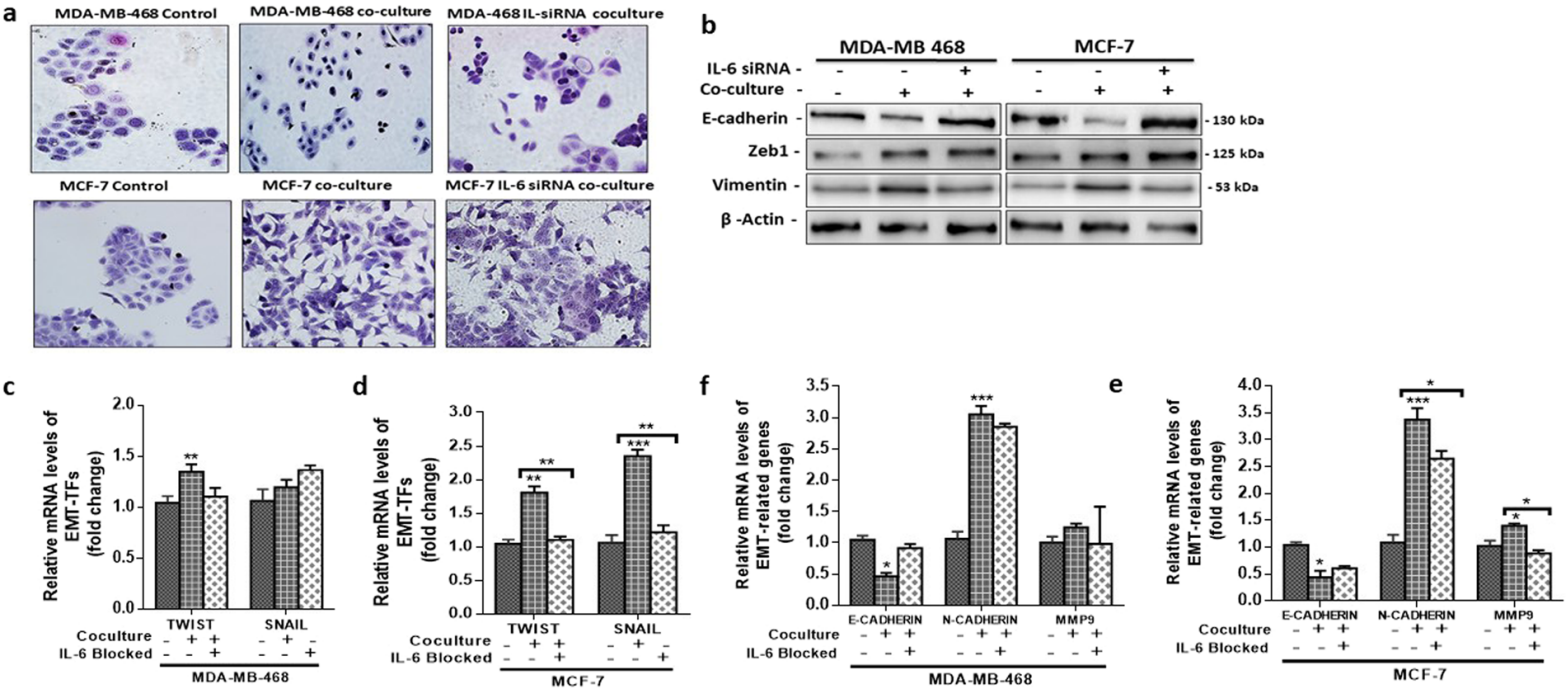
Expression of EMT-related genes was reversed after blocking IL-6 signalling. (**a**) Morphological characteristics of co-cultured breast cancer cell with/without IL-6 blocking and control breast cancer cells. (**b**) Western blot for e-cadherin, vimentin and ZEB1 in co-cultured breast cancer cells with/without IL-6 blocking and in control cells. Full-length blots are presented in Supplementary Fig. 5. (**c**–**f**) Quantitative PCR (qt-PCR) comparing the expression of EMT-TFs and EMT-related genes in co-cultured breast cancer cells with/without IL-6 blocking and control cells. Relative mRNA expression was normalized to GAPDH. All results are representative of 3 independent experiments. (Data indicate mean ± SEM; ***p < 0,001; **p < 0.01; *p < 0.05).

To determine is the IL-6/STAT3 signalling pathways is responsible for the observed EMT-phenotype in co-cultured breast cancer cells. We examined the expression of EMT-associated proteins and genes in breast cancer cells after IL-6 signalling was blocked. Blocking IL-6 signalling did not reverse co-cultured breast cancer cells to their phenotype (Fig. 7a) both MDA-MB-468 and MCF-7 cells remained slightly dispersed and elongated. This may be due to the presence of other adipocyte secreted growth factors such as TGF-β. The expression of EMT-related proteins (E-cadherin, Vimentin and ZEB1) were examined by western blotting after IL-6 signalling was blocked. Blocking IL-6 results in an increase in E-cadherin (Fig. 7b) in both breast cancer cells. The expression of ZEB1 and vimentin was not affected after IL-6 signalling was blocked in both MDA-MB-468 and MCF-7 cells (Fig. 7b). We determined the expression of EMT-related genes after IL-6 signalling was blocked. Our resulted showed changes in gene expression after IL-6 was blocked. The initial decrease in E-cadherin expression in breast cancer cells co-cultured with adipocytes was reversed after IL-6 signalling was blocked in both MDA-MB-468 and MCF-7. The initial upregulation of EMT-TF TWIST in co-cultured breast cancer cells was also reversed after IL-6 signalling was blocked (Fig. 7c,d) in both breast cancer cells. Blocking IL-6 signalling also resulted in SNAIL downregulation in MCF-7 cells (Fig. 7d), whereas in MDA-MB-468 SNAIL was upregulated (Fig. 7c). EMT-related gene expression was also reversed after was IL-6 blocked, E-cadherin was upregulated, whiles N-cadherin and MMP9 were downregulated in both breast cancer cells after IL-6 blocking (Fig. 7e,f). Taking together, these results suggests that IL-6 secreted by adipocytes may be a key regulator of the EMT program in breast cancer cells, vis alteration of EMT-TFs and EMT-related genes expression. The differences in gene expression patterns may be due to the differences in the molecular characteristic of the breast cancer cells as it has been previous reported that induction of EMT occurs through common and/or unique activation of EMT-TFs in breast cancer cells with different characteristics^25^.

## Discussion

Recent studies have begun provide insight into the influence of adipocytes in breast cancer progression particularly in migration, invasion and EMT^14,24^. Several of these studies demonstrate the ability of adipocytes to enhance breast cancer cells motility, migration, invasion and induce EMT^14,24,26^. However, the molecular mechanism by which adipocytes induce EMT and enhance the aggressive characteristics of breast cancer cells has not been fully elucidated. In this study, using the transwell *in-vitro* co-culture system to mimic the breast cancer stromal microenvironment, we show that the presence of mature human adipocytes can enhance the proliferation, migration and invasion capabilities (Fig. 2a–i) in two breast cancer cell lines (MDA-MB-468 and MCF-7) and induce an EMT-phenotype, characterised by decrease expression of E-cadherin and increased expression of vimentin and ZEB1 (Fig. 3b,c). Furthermore, blocking IL-6 signalling with siRNA and neutralising antibody in co-cultured breast cancer cells and adipocyte media, resulted in increased E-cadherin, and attenuation of EMT-related genes such as TWIST and N-cadherin (Fig. 7e,f). Hence, paracrine IL-6 signalling maybe a key regulatory pathway by which adipocytes induce EMT and promote proliferation, migration and invasion in both luminal (MCF-7) and basal (MDA-MB-468) breast cancer cells.

Adipose tissues are major components of the breast cancer microenvironment, they were previous considered as insulating and energy storages units^12,13^, however, recently, their endocrine functions have begun to emerge and have been identified as key players in the tumour microenvironment influencing cancer cell motility, migration and invasion^15,26,27^. Dirat *et al*. (2011) demonstrated that cancer-associated adipocytes enhance the invasion characteristic of breast cancer cells with the emergence of incomplete EMT^13^. Lee *et al*. showed that murine adipocytes condition media induced EMT in breast cancer cells^24^. Collectively, these results suggested that adipocytes can promote migration and invasion through the induction of EMT in different breast cancer cell lines. The potential for adipocytes to enhance breast cancer migration and invasion through the induction of EMT is proposed to occur through the secretion a wide variety of cytokines, growth factors and hormones referred to as adipokines/ adipocytokines^13,16,28,29^. Hence, an understanding of the underlying mechanism of how adipocytes can influence breast cancer cells behaviour and identification of regulatory factors involved may help identify prognostic or therapeutic targets. Various studies have independently shown that growth factors such as TGF-β and IL-6 secreted by stromal cells in the breast cancer microenvironment can influence cancer progression by enhancing migration, invasion and inducing EMT^30–32^. Recent studies have shown that cancer-associated fibroblast through paracrine TGF-β signalling can induce EMT in breast and bladder cancer cells^25,32^. Xie *et al*. also demonstrated that IL-6 can induce EMT and stemness in breast cancer cells^23^. These studies indicate the influence of stromal cells present in the tumour microenvironment on cancer cell migration and invasion. TGF-β and IL-6 are secreted by adipocytes, hence an understanding of how these factors secreted by adipocytes influence breast cancer migration and invasion is required.

The effect of adipocyte on breast cancer cells was tested in a co-culture system, mimicking the breast cancer microenvironment. We observed that co-culture with adipocyte enhance the proliferation, migration and invasion capabilities of MDA-MB-468 and MCF-7 breast cancer cells. The increased aggressive phenotype observed after co-culture may be due to secreted factors by adipocytes or by their direct contact. Indicating that human adipocyte may be active regulators of the proliferation, migration and invasive capabilities of breast cancer cells, mostly likely through the secretion of various growth factors. D’Esposito *et al*. have demonstrated the ability of adipocytes to enhance triple negative breast cancer cell invasiveness and dissemination by secreting CCL5^12^. Our study, confirm previous findings that adipocytes can enhance the aggressive behaviour of breast cancer cells^12,24^. This study also demonstrates that the migratory and invasive capabilities of MCF-7 breast cancer cells which have low metastatic potential can be enhanced after co-culture with adipocytes. Thus, adipocytes in the tumour microenvironment maybe key regulators of the proliferation, migration and invasion of breast cancer cells.

EMT is a feature of metastatic cancer cells, associated with increased invasion and metastatic potential^9^. Epithelial cells undergoing EMT typically have a decrease in the epithelial cell marker E-cadherin expression and an increase in mesenchymal markers vimentin and N-cadherin^6^. Induction of EMT enhance the metastatic capabilities of epithelial cancer cells, various paracrine signals in the microenvironment have been shown to be capable of inducing EMT. It’s well established that cancer-associated fibroblast present in the breast cancer microenvironment can induce EMT through paracrine signalling involving a number of EMT-inducers such as TGF-β and IL-6^25,31,32^. Lee *et al*. also showed that co-culture of breast cancer cells with murine adipocytes can induce an EMT-phenotype^24^.

In addition to the ability of adipocytes to enhance the aggressive phenotype of breast cancer cells, we also show that co-culture with adipocytes also induce an EMT phenotype in breast cancer cells. Our results showed that breast cancer cells co-cultured with human adipocytes had a decreased expression of E-cadherin and an increased expression of vimentin which are key markers for EMT (Fig. 3b,c). Along with the decrease in E-cadherin, co-cultured breast cancer cells had an increase ZEB1 a key markers of EMT-phenotype. The emergence of an EMT-phenotype in co-cultured breast cancer cells may be responsible for the increased migration and invasion of breast cancer cells co-cultured with adipocytes. Thus, adipocytes in the tumour microenvironment maybe capable of inducing EMT in breast cancer cells by similar mechanism as cancer-associated fibroblasts. Induction of EMT is regulated by transcription factors, we analysed the expression of key EMT-TFs after co-culture of breast cancer cells with adipocytes. We observed an increase in the EMT-TFs; TWIST and SNAIL (Fig. 3d,e), thus the EMT-phenotype observed in co-cultured breast cancer cells involved the activation of key transcription factors. However, the regulation of EMT in the different breast cancer cells may occur via a common and unique set of EMT-related genes. Since, SNAIL and MMP9 expression was significantly upregulated in MCF-7 cells but not in MDA-MB-468 cells. The observation that regulation of EMT is by different EMT-related genes in different breast cancer cells has been reported by Yu *et al*.^25^. Thus, adipocytes induced aggressive behaviour in breast cancer cells may occur through the induction of EMT, regulated by the upregulation of key EMT-TFs and EMT-related genes.

In the tumour microenvironment, induction of EMT in cancer cells can occur through various paracrine signals. To identify a common paracrine signalling mechanism by which adipocyte induced EMT in breast cancer cell occur, we determined the expression of TGF-β and IL-6 (known regulator of EMT in breast cancer cells) in breast cancer cells and adipocytes after co-culture. IL-6 is a pleotropic cytokine associated with several aspects of tumour progression including EMT, stemness, angiogenesis and therapeutic resistance^20–22^. Increased IL-6 has been reported in serum of breast cancer patients and associated with poorer prognosis^33^. Thus, IL-6 has emerged as a potential target for therapy and various agents have been developed that target IL-6. We found IL-6 and its downstream target phosphorylated STAT3 to be significantly activated in both breast cancer cells after co-culture. Increased expression of IL-6 resulted in phosphorylation of STAT3 and its nuclear accumulation in co-culture breast cancer cell. STAT3 phosphorylation and activation is known has been shown to enhance breast cancer migration through the induction of gene involved with EMT, such as SNAIL, MMP9 and TWIST and also induced autocrine secretion of IL-6^23,31,34^. Blocking of IL-6 receptor with siRNA in breast cancer cells and neutralization of secreted IL-6 by adipocytes with neutralising IL-6 antibody, decreased the STAT3 phosphorylation and nuclear localization with decreased proliferation, migration and invasion capabilities of both breast cancer cell after co-culture (Fig. 5h–j). This results demonstrates a role for IL-6/STAT3 in the interaction between adipocytes and breast cancer cells. Hence, paracrine IL-6 signalling may be a key regulator of adipocytes induced proliferation, migration and invasion in breast cancer cells, through phosphorylation and nuclear localization of STAT3. Activation of STAT3 may regulate the expression EMT-related genes in co-cultured breast cancer cells. This observation is consistent with previously published data on the ability of adipocytes to influence breast cancer cell proliferation, migration and invasion and also confirms the role of IL-6 in tumour invasion and migration^14,24,26^. The inability of IL-6 blocking to completely inhibit proliferation, migration and invasion indicates the possibility for other growth factors secreted by adipocytes to be involved in the process.

Furthermore, blocking IL-6 signalling altered of EMT-TFs and EMT-related gene expression. Blocking IL-6 resulted in E-cadherin upregulation in both breast cancer cells, while the expression TWIST and N-cadherin was downregulated. These results clearly show that blockage of IL-6 signalling does not only affect the aggressive behaviour and but also alters the expression of EMT-related genes. This alteration in EMT-TFs and EMT-related gene expression may occur through the activity of STAT3. The results from blocking IL-6 signalling further confirmed our observation that the EMT-program is regulated by common and unique EMT-related gene expression patterns in different breast cancer cells. The initial increase in SNAIL expression in MCF-7 was reversed after IL-6 blockage, however SNAIL expression increased in MDA-MB-468 cell. These results indicates that paracrine IL-6/STAT3 signalling maybe a key regulator of adipocyte induced EMT in breast cancer cells and occur via induction of unique EMT-related genes in different breast cancer cells^35,36^. Recent studies by Lee *et al*.^24^ supports our findings, that adipocytes may induce EMT in breast cancer cells, though we may be the first to suggest paracrine IL-6/STAT3 to be involved in adipocytes induced development of EMT in MDA-MB-468 and MCF-7 cells.

We propose that adipocytes can induce an EMT program in MCF-7 and MDA-MB-468 breast cancer cell, resulting in enhanced proliferation, migration and invasion capabilities in these breast cancer cells. The induction of EMT in these cells by adipocytes occur by the well-established IL-6/STAT3 signalling pathway. This observation maybe relevant for future therapies targeting components in the breast cancer microenvironment, as patients can be stratified based on the infiltration of adipose tissue into breast tumours. Therapeutic agents targeting the IL-6/STAT3 pathway may have potential in this subset of patients. Further studies are required to completely elucidate the molecular mechanisms by which adipocytes influence breast cancer progression. These studies may focus on understanding how breast cancer associated adipocytes differ in gene expression profile from normal adipocytes and if adipocytes contribute to the development and maintenance of breast cancer stem cells.

In conclusion, this study demonstrates the ability of human adipocytes to enhance proliferation, migration and invasion in MDA-MB-468 (basal) and MCF-7 (luminal) breast cancer cells. Our data provide evidence for the first time that the IL-6/STAT3 signalling pathway may be key regulatory pathway by which adipocytes affect breast cancer cell behaviour. We also show that adipocytes also induce an EMT phenotype regulated by expression of key EMT-related genes in breast cancer cells.

## Methods

### Isolation, immortalization and differentiation of human preadipocytes into mature adipocytes

To prepare immortalized preadipocytes, white adipose tissue was isolated from by-product of human patients with colon cancer as described by Lee *et al*.^37^. Briefly isolated adipose tissues were washed with PBS, minced, and digested with type II collagenase (2 mg/ml) in Krebs-Ringer bicarbonate buffer (KRBB) containing 10 mM HEPES (pH 7.4), and 3% BSA for 1 hr at 37 °C. Following passage through a 300 μm mesh and centrifugation floating adipocytes were collected by aspiration. Pellets containing the stromal vascular (SV) fraction were incubated in red blood cell lysis buffer for 5 min at room temperature. Passed through a 40 μm mesh, and primary preadipocytes collected by centrifugation at 500 g for 5 min. The resultant cell preparations were subjected to immunostaining or flow cytometry. This study was proved by the Institutional Review Board. Primary preadipocytes are expanded in growth medium Dulbecco’s modified Eagle’s medium (DMEM; Welgene, Inc. Seoul, Korea) with 10% fetal bovine serum (FBS; Gibco^TM^, Life Technologies, Carlsbad, CA, USA) and 1% penicillin-streptomycin (Gibco^TM^). Primary adipocytes were differentiated at passage 2 in adipogenic differentiation medium [DMEM with 10% FBS and 1% Penicillin-streptomycin supplemented with 2.5 mM isobutylmethylxanthine (IBMX) (Sigma-Aldrich, St Louis, MO, USA), 1 μM dexamethasone (Sigma-Aldrich), 1 μg/ml insulin (Sigma-Aldrich)] for 12 days. Cells were subsequently maintained in medium containing insulin for up to 2 weeks. Matured adipocytes were used when about 70% of cells were differentiated into matured adipocytes and was confirmed by Oil Red O staining (Sigma-Aldrich). All methods and experimental protocols using human tissue were carried out in accordance with relevant guidelines and regulations approved by the Institutional Review Board of Severance Hospital, Yonsei University Health System (4-2014-0054). The informed consent was acquired from patients and all methods were carried out in accordance with relevant guidelines and regulations of Institutional Review Board of Board of Severance Hospital, Yonsei University Health System.

### Cell culture of breast cancer cells

The human breast cancer cell lines MDA-MB-468 (Estrogen receptor (ER) negative, Progesterone receptor (PR) negative and Human epidermal growth factor receptor-2 (HER2) negative, basal type) was cultured in DMEM mixed with F12 (DMEM/F12; Welgene) supplemented with 10% FBS and 1% penicillin-streptomycin and MCF-7 (ER, PR positive and HER2 negative, luminal type) was cultured in DMEM/F12 supplemented with 10% FBS, 1% penicillin-streptomycin and 0.1 mg/ml insulin, in a humidified 5% CO_2_ atmosphere. Cultured cells at 70–80% confluence were used in experiments.

### Co-culture, cell migration and invasion assay

A total of 5 × 10^5^ breast cancer cells were seeded in the upper chamber of a Costar Transwell culture plates (0.4 μm pore size; Corning Inc., Corning, NY) and co-cultured with or without differentiated adipocytes in the bottom well for 48 hours. The effect of adipocyte on breast cancer cell migration were evaluated with transwell insert (8 μm pore size; Corning Inc.) and invasion with matrigel-coated transwell insert (1.0 mg/ml matrigel; BD Biosciences, Bedford, MA, USA). Breast cancer cells are maintained in serum free media overnight and 5 × 10^4^ cells in 300 μl of serum free media added to the upper chamber with mature adipocytes in 10% FBS media or 10% FBS media without adipocytes in the bottom chamber. Cells were cultured in humidified 5% CO_2_ atmosphere for an appropriate time, non-invading cells were removed with a cotton swap and invading cells were fixed and stained by the Diff-Quik kit (Sysmex, Seoul, Korea). The number of invading and migrating cells was counted under a microscope in five random fields of each membrane at x100 magnification.

### Cell proliferation assay

To determine the cell proliferation rate, breast cancer cell co-cultured with and without adipocytes for 48 hours, are trypsinized and 0.25 × 10^4^ cells in culture media was seeded into a 96 well plate and maintained for the appropriate length of time (24, 48, 72 and 96 hours, all experiments are performed in triplicate. After each time period, 10 μl of CCK-8 solution (Dojindo, Kumamoto, Japan) was added to each well and incubated for 2 hours and optical density was measured at 450 nm in a microplate reader (Tecan Group limited, Männedorf, Switzerland).

### Wound-healing assay

Breast cancer cells co-cultured with and without adipocytes were plated in a six well plate at 5 × 10^5^ and allowed to grow into a monolayer. At 90% confluence, complete media is replaced with serum free media and cells are cultured overnight until a monolayer is formed. Using a sterile 100 μl pipette tip a linear scratch was made on the cell monolayer. Cells are rinsed with PBS and fresh complete media added. Photomicrograph was taken of migrated cells at 0 and 48 hours at x100 magnification. The captured images were analysed using the TScratch software.

### RNA extraction and Reverse transcription-quantitative PCR (qtPCR)

Total RNA of cells cultured with and without adipocytes was isolated using the RNeasy Kit (Qiagen, Valencia, CA, USA) following manufacturer’s instruction. Real time PCR was performed with 50 ng of RNA using the One Step SYBR PrimeScript^TM^ RT-PCR kit (Takara Shuzo Co., Japan) according to the manufacturer’s instruction and analysed with the StepOne Plus Real-time PCR system (Applied Biosystems, Foster City, CA, USA). All reactions were performed in triplicate; with the housekeeping gene glyceraldehyde 3-phosphate dehydrogenase (GAPDH) as an internal control mRNA. All primers were initially evaluated for efficiency using the Relative standard curve and the relative gene expression evaluated by comparative CT method (2^−∆∆CT^). Primer sequences are listed in Table 1.

**Table 1:** Primer sequences.

Primer	Sequence
IL-6	CCAGCTATGAACTCCTTCTC GCTTGTTCCTCACATCTCTC
TGF-β	CTAATGGTGGAAACCCACAACG TATCGCCAGGAATTGTTGCTG
SNAIL	CACCTCCAGACCCACTCAGAT CCTGAGTGGGGTGGGAGCTTCC
MMP9	CCTGCCAGTTTCCATTCATC GCCATTCACGTCGTCCTTAT
TWIST	CCACGCTGCCCTCGGACAAG CCAGGCCCCCTCCATCCTCC
N-Cadherin	GCGTCTGTAGAGGCTTCTGG GCCACTTGCCACTTTTCCTG
E-Cadherin	CTGAGAACGAGGCTAACG TTCACATCCAGCACATCC
STAT3	TGAGACTTGGGCTTACCATTGGGT TCTTTAATGGGCCACAACAGGGCT
GAPDH	ACCCACTCCTCCACCTTTGA CTGTTGCTGTAGCCAAATTCGT

### Western blotting

Harvested cells were lysed in RIPA buffer (150 mM sodium chloride, 1% triton X-100, 1% sodium deoxycholate, 0.1% SDS, 50 mM Tris-HCl, pH 7.5 and 2 mM EDTA) (GenDEPOT, TX, USA) containing 1% protease inhibitor cocktail (GenDEPOT). 20 μg of each sample is separated by SDS-PAGE and transferred to a nitrocellulose membrane (GE Healthcare, Chalfont St Giles, UK). Western blotting was performed as previously described by Liu *et al*.^38^ with primary antibodies to mouse anti-E-cadherin 1:1000 (Cell Signalling, Danvers, MA, USA), rabbit anti-Vimentin 1:500 (Abcam, Cambridge, UK), rabbit anti-Zeb1 1:500 (Sigma), rabbit anti-IL-6 1:1000 (Abcam), rabbit anti-STAT3 1:500 (phosphor Y705: Abcam), mouse anti-STAT3 1:1000 (Cell signalling), rabbit anti-α-lamin 1:1000 and goat anti-β-actin 1:5000 (Santa Cruz Biotechnology, Santa Cruz, USA). All primary antibodies were diluted in 5% Bovine Serum Albumin (BSA) in Tris-buffered saline (TBS) containing 0.1% Tween-20 (TBST) and incubated overnight at 4 °C. Secondary antibody included goat anti-rabbit IgG-HPR 1:5000 (Santa Cruz Biotechnology), goat anti-mouse IgG-HPR 1:2000 (Santa Cruz Biotechnology) and rabbit anti-goat IgG-HPR (GenDepot, TX, USA). Protein bands were visualised using enhanced chemiluminescence reagents (Western Lighting Plus, PerkinElmer, USA).

### Immunofluorescence staining

Cells were seeded on cover slide placed in co-culture insert and cultured with/without adipocytes for 48 hr. Cells was rinsed in PBS and fixed with 4% paraformaldehyde, permeabilized with 0.2% Triton X-100 and stained with appropriate primary antibodies. For double staining experiments, antibodies were diluted together and incubated with cells overnight at 4 °C. Goat Anti-Rabbit IgG (Alexa Fluor 647) and Goat Anti-Mouse IgG (Alexa Fluor 488) antibodies (Abcam) were used as secondary antibodies. Counter staining of cell nuclei was performed using DAPI (Invitrogen, Carlsbad, CA, USA). Stained cells were visualized using the ZEISS LSM 710 microscope (ZEISS, Germany). Antibodies used included mouse anti-E-cadherin 1:500 (Cell Signalling, Danvers, MA, USA), rabbit anti-Vimentin 1:500 (Abcam, Cambridge, UK), mouse anti-STAT3 (dilution 1:200) (Cell Signalling Technology) and rabbit anti-P-STAT3(Y705) (dilution 1:500) (Abcam).

### Transfections and Luciferase reporter assays

MDA-MB-468 and MCF-7 breast cancer cells were seeded at 1 × 10^6^ cells in a 100 mm dish in antibiotic free media overnight and transfected with 20 nM IL-6R siRNA from On-target Plus Smart Pool (Dharmacon, Lafayette, CO, USA) or control siRNA (Dharmacon) as previous described by Rosner e*t al*.^39^ using lipofectamine RNAiMAX reagent (Invitrogen). Experiments with siRNA transfected cells were conducted 72 hrs after transfection and co-culture with human adipocytes. Differentiated human adipocytes was also transfected with 20 nM IL-6R siRNA from On-target Plus Smart Pool (Dharmacon, Lafayette, CO, USA). The transfection efficiency was determined by western blot analysis.

MDA-MB-468 and MCF-7 were transfected with STAT3 reported plasmid (Cignal Lenti STAT3 Reporter, QIAGEN, Hilden, Germany) using SureENTRY transduction reagent (Qiagen). Stable STAT3 reporting cells were selected with 400 µg/ml of puromycin (Sigma) over 10 days to generate a stable STAT3 reporter cell line for MDA-MB-468 and MCF-7. STAT3 promoter activity was determined by Promega Dual-Luciferase reporter assay system (Promega corporation, Madison, USA) and luciferase activity measured in the Tecan™ microplate-Luminometer (Tecan Group limited, Männedorf, Switzerland). The constitutively expressed non-inducible Renilla luciferase activity served as internal control for normalizing transfection efficiencies.

### Adipocytes IL-6 Neutralisation

To differentiated human adipocytes in 6-well plate, 400 ug/ml IL-6 antibody (Abcam) was added to adipocytes media to neutralise IL-6 secreted by adipocytes.

### Statistical analysis

Data were analysed and graphs plotted with Graphpad Prism version 6 software (GraphPad Inc.). Student’s t-test was used to compare differences between two groups and multiple analysis was performed using analysis of variance (ANOVA). Multiple analysis of groups was checked for after ANOVA using Bonferroni’s multiple comparison test. Statistical significance was defined as P < 0.05.

## Electronic supplementary material

Figure 1, Figure 2, Figure 3, Figure 4, Figure 5
