# Author Correction: Interleukin-6/STAT3 signalling regulates adipocyte induced epithelial-mesenchymal transition in breast cancer cells

**DOI:** 10.1038/s41598-020-69834-x

**Published:** 2020-07-29

**Authors:** Jones Gyamfi, Yun-Hee Lee, Minseob Eom, Junjeong Choi

**Affiliations:** 1 College of Pharmacy, Yonsei Institute of Pharmaceutical Sciences, Yonsei University, Incheon, Korea; 2 Department of Pathology, Yonsei University Wonju College of Medicine, Wonju, Korea

Correction to: *Scientific Reports* 10.1038/s41598-018-27184-9, published online 11 June 2018.

This Article contains errors.

In Figure 5C, the image for pSTAT3 stain for MCF-7/IL-6 siRNA is incorrect. In addition, the images for MCF-7 co-culture are incorrect. In addition, the figure legend for Figure 5 does not correspond to the arrangement of panels. The correct Figure 5 appears below as Figure 1, along with a revised figure legend.

**Figure 1:**
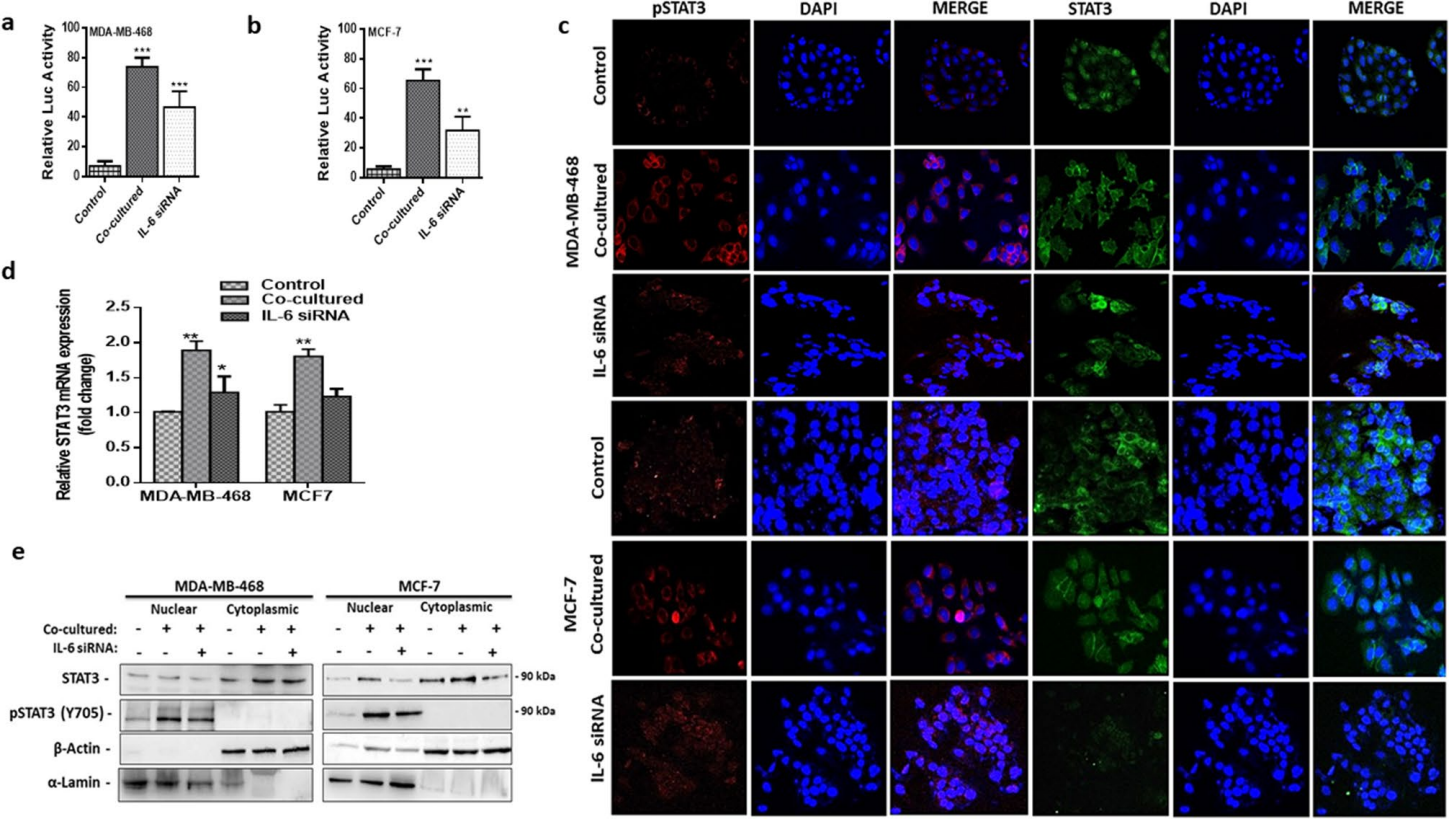
(**a**,**b**) Relative luciferase activity in breast cancer cells with STAT3 luciferase reporter plasmid, with/without human adipocytes and after IL-6 was blocked. STAT3 luciferase activity was measured after 48 hours. (**c**) STAT3 phosphorylation and nuclear localization in co-cultured human breast cancer cells with/without IL-6 neutralization and in control cells assessed by immunofluorescence staining. (**d**) Quantitative PCR comparing the expression of STAT3 mRNA in co-cultured breast cancer cells with/without IL-6 blocking and in control cells. Relative mRNA expression was normalized to GAPDH. (**e**) Representative western blot analysis of pSTAT3 expression in cytoplasmic and nuclear fraction of co-cultured breast cancer with/without IL-6 neutralization and in control breast cancer cells.

This error affects Supplementary Figure 4, for which the pSTAT3 stain of MCF-7/IL-6 siRNA is incorrect. The correct Supplementary Figure 4 appears below as Figure 2.

**Figure 2:**
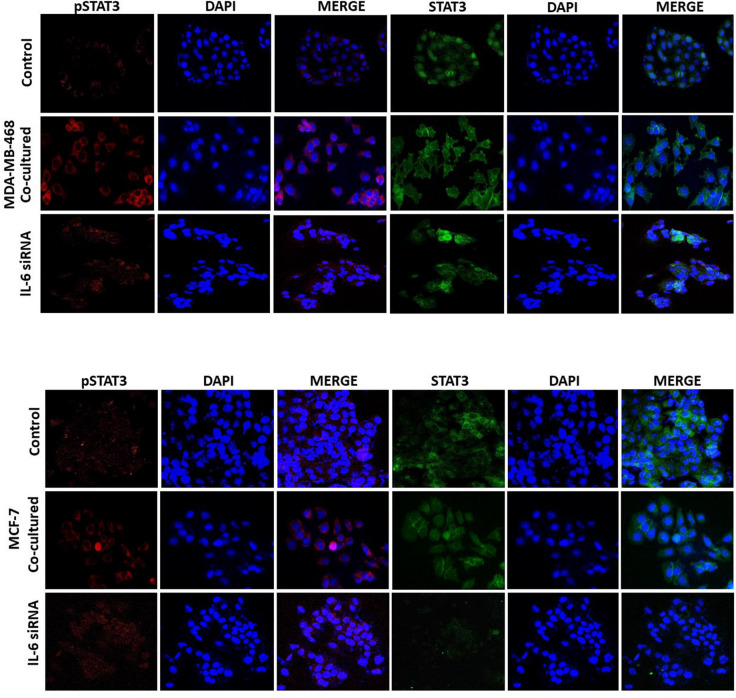
.

