# Supplementary material for: Interleukin-6/STAT3 signalling regulates adipocyte induced epithelial-mesenchymal transition in breast cancer cells
Source: Sci Rep. 2018 Jun 11;8:8859. doi: 10.1038/s41598-018-27184-9 (PMC5995871; doi:10.1038/s41598-018-27184-9)
Supplement: Supplementary file 1 — Figure 1, Figure 2, Figure 3, Figure 4, Figure 5 [file 41598_2018_27184_MOESM1_ESM.docx]

**Supplementary Information**

**Interleukin-6/STAT3 signalling regulates adipocyte induced epithelial-mesenchymal transition in breast cancer cells.**

^1^Jones Gyamfi, ^1^ Yun-Hee Lee, ^2^Minseob Eom ^1^Junjeong Choi

^1^ College of Pharmacy, Yonsei Institute of Pharmaceutical Sciences, Yonsei University, Incheon, Korea

^2^ Department of Pathology, Yonsei University Wonju College of Medicine, Wonju, Korea

**Corresponding author:**

Junjeong Choi M.D., Ph. D.

College of Pharmacy, Yonsei Institute of Pharmaceutical Sciences, Yonsei University, Incheon, Korea

Veritas Hall D 306, 85 Songdogwahak-ro,
Incheon, 21983, Republic of Korea

Phone: +82-32-749-4521

Fax: +82-32-749-4105

E-mail: [junjeong@yonsei.ac.kr](mailto:junjeong@yonsei.ac.kr)


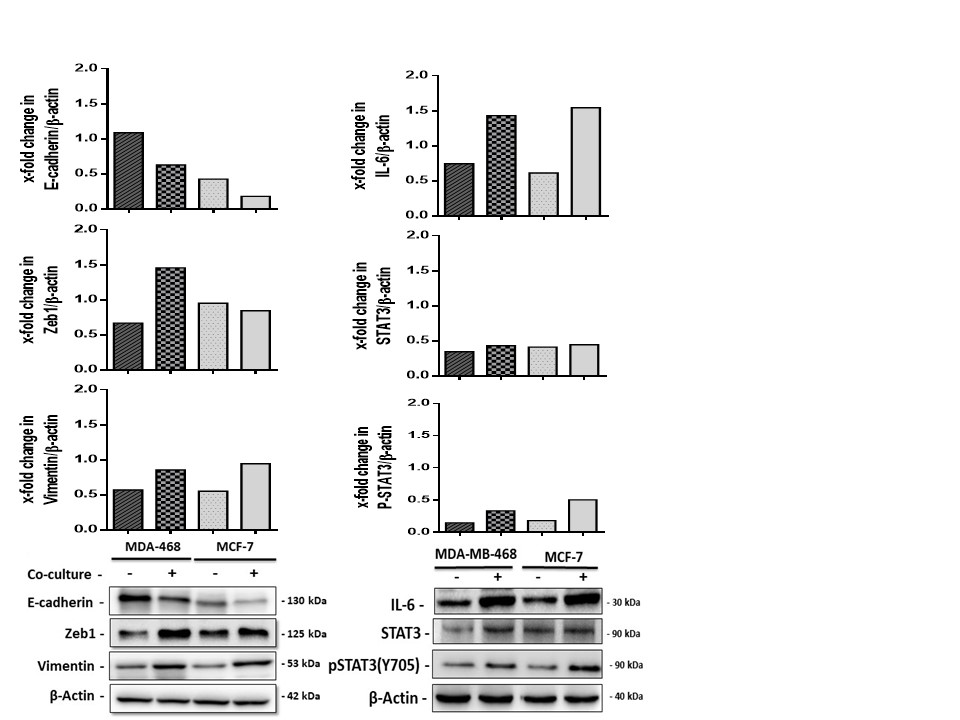


**Supplementary Figure 1: Adipocytes induces an EMT-phenotype in co-cultured breast cancer cells.**

Representative Western blot and quantification showing levels of E-cadherin, Zeb1 and vimentin in co-cultured breast cancer cells.


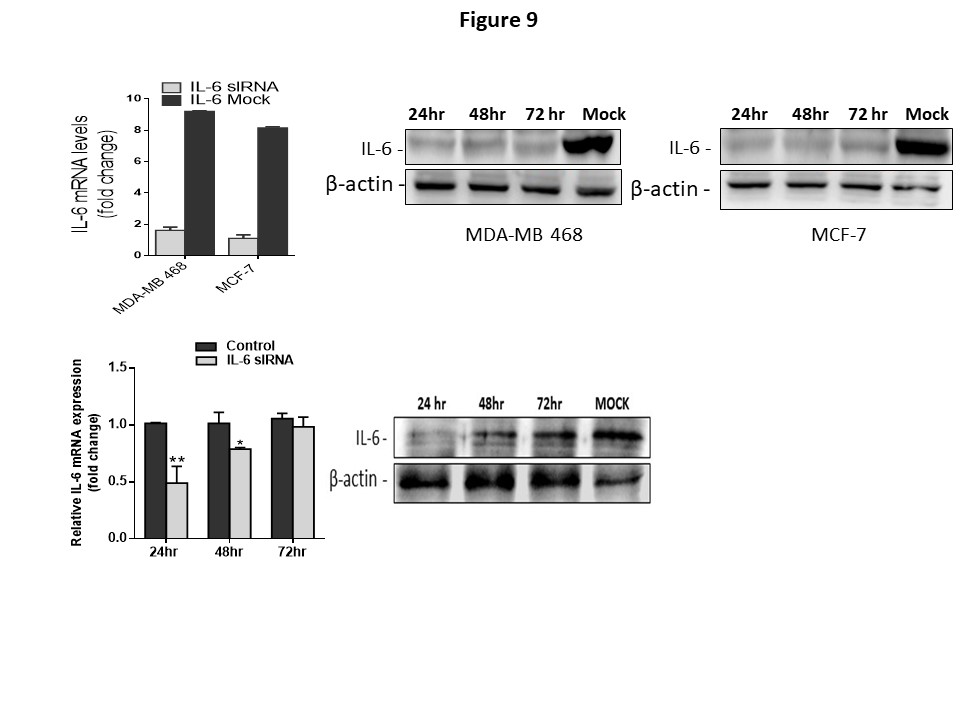


**Supplementary Figure 2: Inhibition of IL-6R by siRNA in breast cancer cells and in differentiated human adipocytes.**


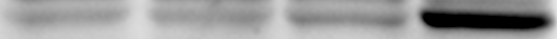

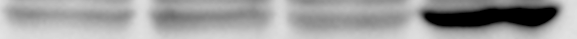


MCF-7

MDA-MB 468


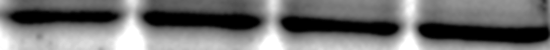


IL-6 -

**24hr 48hr 72 hr Mock**


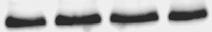


**24hr 48hr 72 hr Mock**

QT-PCR quantification and Western blot analyses of IL-6 expression in breast cancer cells and differentiated human adipocytes after treatment with IL-6R siRNA. Expected band size for IL-6 is 30kDa, blots show inhibition of IL-6 over 96 hours after treatment with siRNA in breast cancer cells. Inhibition of IL-6R in human adipocytes was effective within 24 hours. Beta-actin expected molecular weight/size is 42 kDa. Relative IL-6 mRNA expression was normalized to GADPH (Data indicate mean ± SEM; ***p < 0,001; **p < 0.01; *p < 0.05).


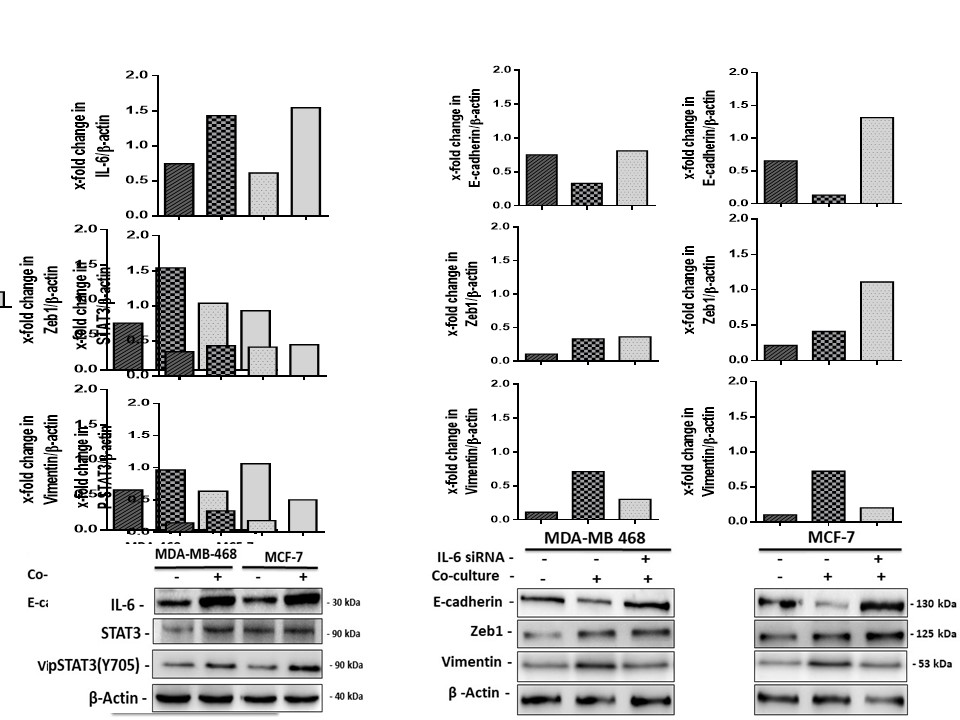


**Supplementary Figure 3: Adipocytes induces an EMT-phenotype in co-cultured breast cancer cells is reversed by IL-6 blockage.**

Representative Western blot and quantification showing levels of E-cadherin, Zeb1 and vimentin in co-cultured breast cancer cells.


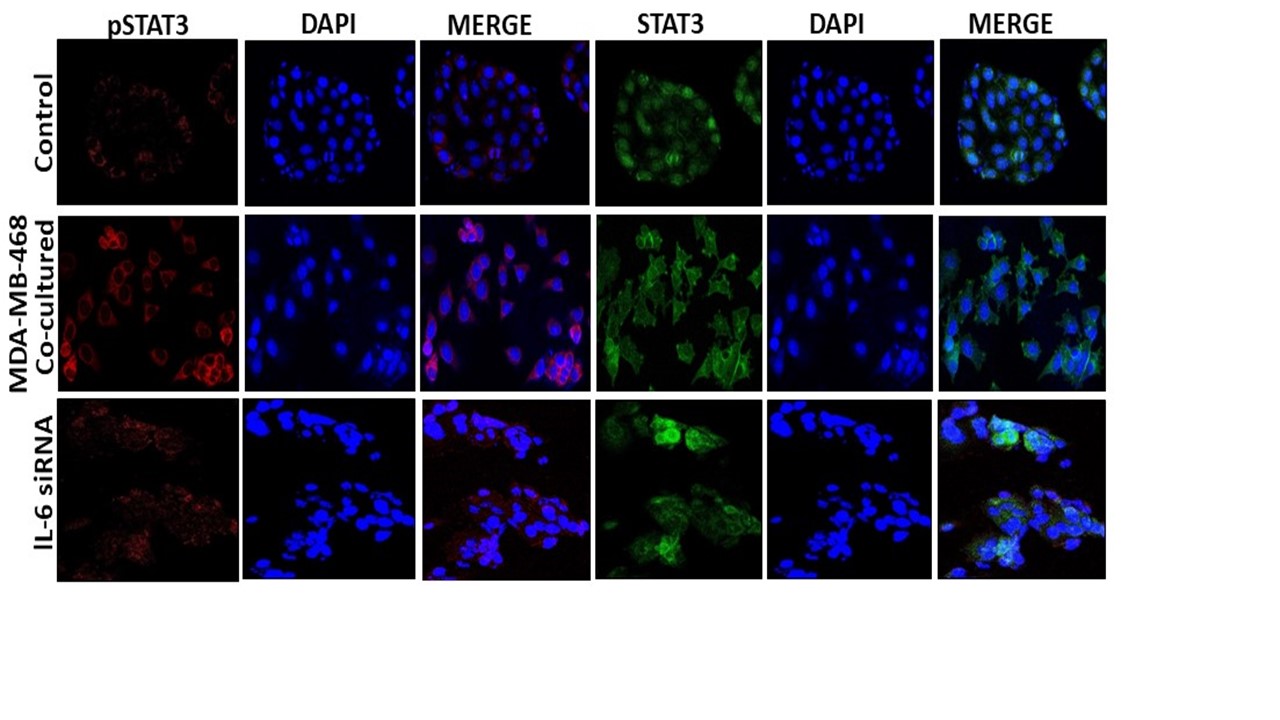


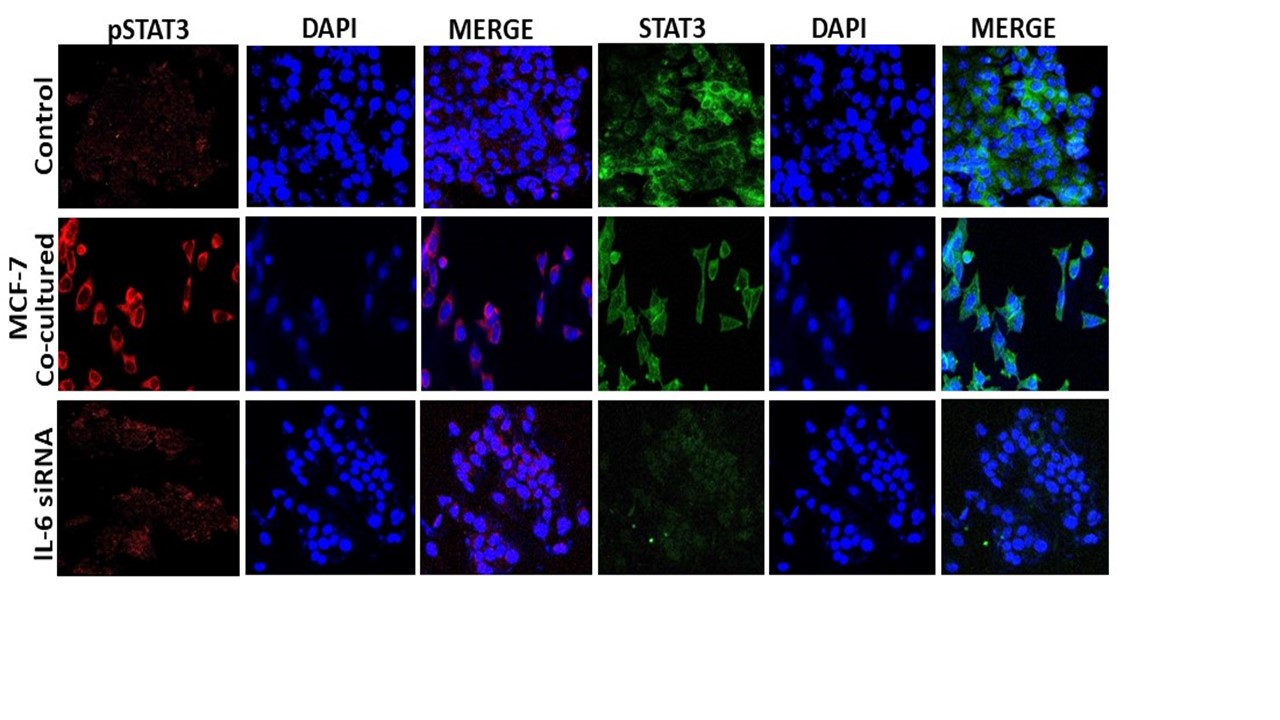


**Supplementary Figure 4: Nuclear localization of pSTAT3 after co-culture of breast cancer cells with human adipocytes.**

Representative confocal images of immunofluorescent stainings of STAT3 (green) and P-STAT3 (red) co-stained with nuclear marker DAPI (blue) in breast cancer cells cultured with adipocytes.


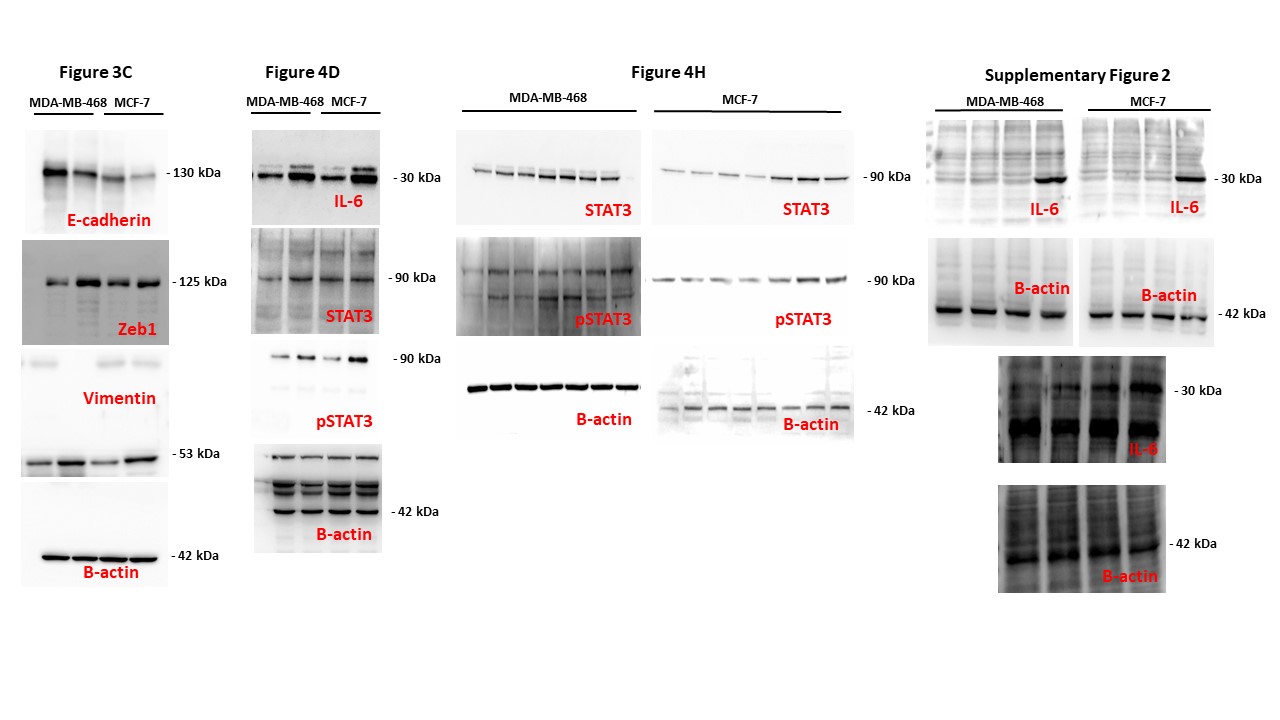

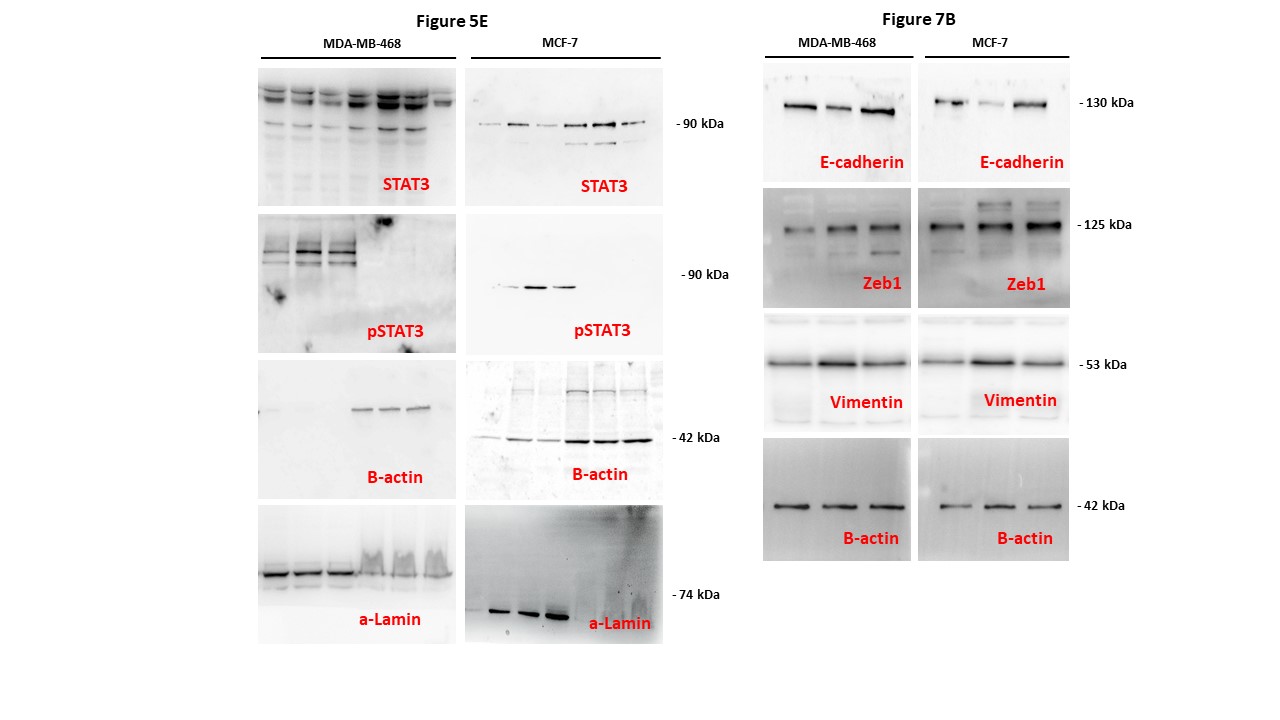


**Supplementary Figure 5: Uncropped scans of western blot displayed in Fig. 3C, 4D, 4H 5E, 7B and supplementary fig 2.**
